# Estimating COVID-19 cases in Puerto Rico using an automated surveillance system

**DOI:** 10.3389/fpubh.2022.947224

**Published:** 2022-08-04

**Authors:** Marijulie Martinez-Lozano, Rajendra Gadhavi, Christian Vega, Karen G. Martinez, Waldo Acevedo, Kaumudi Joshipura

**Affiliations:** ^1^Center for Clinical Research and Health Promotion, University of Puerto Rico Medical Sciences Campus, San Juan, Puerto Rico; ^2^B.J. Medical College, Ahmedabad, Gujarat, India; ^3^Telecommunication Bureau, San Juan, Puerto Rico; ^4^Harvard T.H. Chan School of Public Health, Boston, MA, United States

**Keywords:** COVID-19, surveillance, Puerto Rico, symptom-based model, epidemiology

## Abstract

Due to concerns regarding limited testing and accuracy of estimation of COVID-19 cases, we created an automated surveillance system called “Puerto Rico Epidemiological Evaluation and Prevention of COVID-19 and Influenza” (PREPCOVI) to evaluate COVID-19 incidence and time trends across Puerto Rico. Automated text message invitations were sent to random phone numbers with Puerto Rican area codes. In addition to reported COVID-19 test results, we used a published model to classify cases from specific symptoms (loss of smell and taste, severe persistent cough, severe fatigue, and skipped meals). Between 18 November 2020, and 24 June 2021, we sent 1,427,241 messages, 26.8% were reached, and 6,975 participants answered questions about the last 30 days. Participants were aged 21–93 years and represented 97.4% of the municipalities. PREPCOVI total COVID-19 cases were higher among women and people aged between 21 and 40 years and in the Arecibo and Bayamón regions. COVID-19 was confirmed, and probable cases decreased over the study period. Confirmed COVID-19 cases ranged from 1.6 to 0.2% monthly, although testing rates only ranged from 30 to 42%. Test positivity decreased from 13.2% in November to 6.4% in March, increased in April (11.1%), and decreased in June (1.5%). PREPCOVI total cases (6.5%) were higher than cases reported by the Puerto Rico Department of Health (5.3%) for similar time periods, but time trends were similar. Automated surveillance systems and symptom-based models are useful in estimating COVID-19 cases and time trends, especially when testing is limited.

## Introduction

In Puerto Rico (PR), the first COVID-19 case was identified on 13 March 2020 (1), soon after a series of earthquakes in January 2020, and while PR was still recovering from Hurricane Maria (2017). A lockdown was declared with restrictions varying over time 2 days after the first case was detected in PR (2, 3). The executive orders following the Centers for Diseases Control and Prevention (CDC) guidelines to reduce transmission included enforcement of non-pharmaceutical interventions (NPIs) and a 14-day quarantine requirement for positive COVID-19 cases (4). Since the government's responses to previous disasters had not been adequate, Puerto Ricans were wary about the government's ability to control the pandemic. Despite lockdowns and mass media communications, many people were not convinced of the serious consequences of COVID-19 and/or did not agree with implementing the measures to reduce the risk of infection. This may be partly due to the public receiving conflicting information from PR government, federal agencies, mass media, social media, workplace, and health professionals.

In April 2020, PR had among the lowest testing rates per capita in the United States, with only 4,539 tests run in a population of 3.2 million (5). Importantly, there were concerns about the quality of the tests purchased by the government, which were not approved by the U.S. Food and Drug Administration (6). There were also difficulties with case registration, resulting in many cases counted two times and many missed (7) due to the lack of centralization and standardization of case counting. Since there were many concerns about the Puerto Rico Department of Health (PRDOH) management of COVID-19, some municipalities decided to take charge of case reporting and tracking starting in April 2020 (8). As each municipality managed their own statistics and reports, there was no centralized database of cases until 2021. Hence, the inaccuracy of the reports of cases and the lack of population-based estimates in Puerto Rico were major concerns.

There was a shortage of COVID-19 tests during the earlier stages of the pandemic in the United States (9). Strict criteria were established to qualify for testing in PR (5), limiting who could get tested both for tests offered free of cost as well as tests paid out of pocket. For example, tests in PR were prioritized for people who had traveled or had prolonged contact with reported cases (10). In addition, many people could not get tested due to the limited number of laboratories offering the test and misinformation about the costs to individuals. Low testing resulted in a low number of diagnosed COVID-19 cases. Subsequently, testing was more readily accessible, with the government having multiple sites of free antigen and molecular testing around the island. Since the COVID-19 vaccine became available in December 2020, testing dropped in PR (11) (e.g., average: 9,492 PCR tests on 5 December 2020, vs. 2,936 on 26 November 2021), as well as in the United States overall (12).

Many people who had COVID-19 were asymptomatic. Among people with COVID-19-related symptoms, many could not or chose not to get tested, and it was important to account for the undiagnosed and/or probable cases to the extent possible to get better estimates. Given the gaps described above, we conducted an automated population-based epidemiological surveillance across PR. We assessed COVID-19 testing, diagnosis, and symptoms over time, and used both tests and symptoms to classify cases. This study aims to report the COVID-19 incidence rates and test positivity rates in samples representative of adults living in Puerto Rico over time and by sociodemographic factors and to compare the rates from our surveillance and PR government estimates (two sources with two different approaches). In addition, we evaluate associations between changes in COVID-19 incidence, test positivity, testing rates, and a few key factors to understand their interrelationships.

## Methods

We created an automated surveillance system named “Puerto Rico Epidemiological Evaluation and Prevention of COVID-19 and Influenza” (PREPCOVI) to assess COVID-19 and influenza symptoms, diagnoses, and testing. Computer algorithms were used to generate monthly lists of 200,000 random phone numbers for Puerto Rican area codes to recruit representative samples of adults living in PR. From 18 November 2020 through 24 June 2021, automated text messages were sent to randomly generated phone numbers, inviting adults aged 21 years or over to participate. A link to the initial 5-min survey was included. Completion of the questionnaire implied the participants' consent. The study was approved by the University of Puerto Rico Medical Sciences Campus Institutional Review Board.

Information was collected on key sociodemographic factors (questionnaire included as Supplementary material). Questions to assess COVID-19 and influenza symptoms and diagnoses pertained to the last 30 days before the survey, and since the lockdown in PR (16 March 2020). Participants reporting COVID-19 diagnoses were asked to specify the type of test to classify cases as confirmed (molecular test/PCR) or probable (antigen or serological test); those who did not know the type of test were classified as probable cases. Since testing was a challenge since the beginning of the pandemic (5, 9), it was important to also identify probable cases from symptoms. We identified additional probable COVID-19 cases using a published model. The model used a combination of specific key symptoms (loss of smell and taste, severe persistent cough, severe fatigue, and skipped meals) and adjusted for sex and age to predict probable COVID-19 infection (13).

At the end of the initial survey, participants were asked if they wanted to participate in one of three additional modules. These modules assessed (1) risk and preventive factors and management of COVID-19; (2) knowledge, attitudes, and practices toward COVID-19; or (3) social, economic, and mental health impact of the pandemic. Upon completion, participants were automatically provided electronic health promotion materials tailored to the module they completed, their COVID-19, and vaccination status.

We evaluated the monthly and cumulative distribution of COVID-19 cases overall and by age group, sex, and location. To validate our accuracy of case classification, we compared reported symptoms individually by COVID-19 case status. To help understand real trends vs. trends due to changes in testing, we also evaluated time trends in COVID-19 testing and the positivity rate in PR. We compared PREPCOVI monthly data with PRDOH data to assess consistency and to understand the impact of different methodologies on the estimates. PRDOH data were downloaded from the official site of PRDOH on 14 October 2021; we only included people aged 21 years or older. Since PREPCOVI started on November 18 and the surveys asked about the last 30 days, for comparison we included PRDOH cases since 18 October 2020 (to be consisted in the PREPCOVI study period as well as 30 days before PREPCOVI started). We standardized PRDOH and PREPCOVI data by age and sex using the age and sex distribution for the 2019 Census population. Analysis was conducted using the STATA statistical software version 16.

## Results

**Figure 1:**
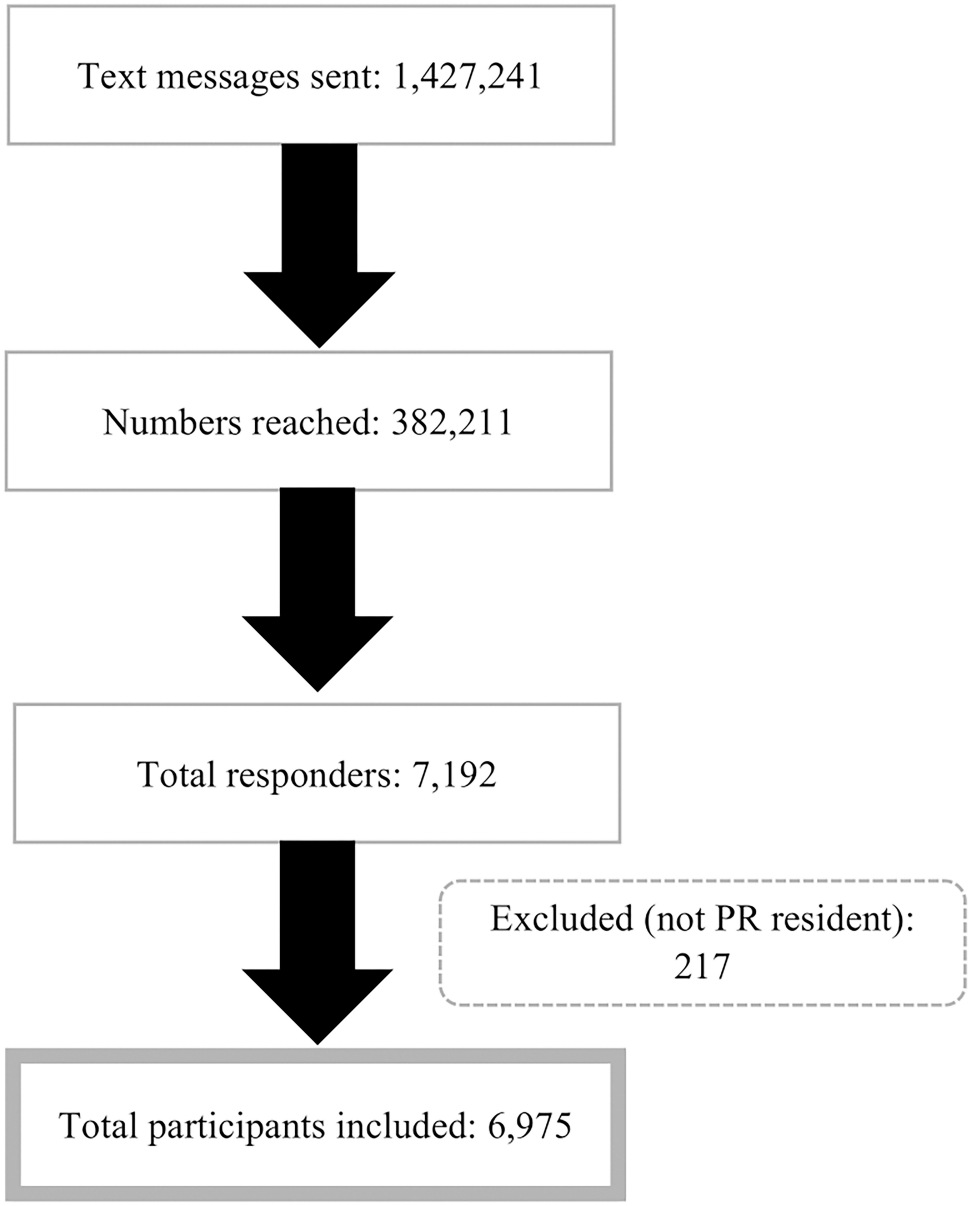
Recruitment of PREPCOVI participants.

**Figure 2:**
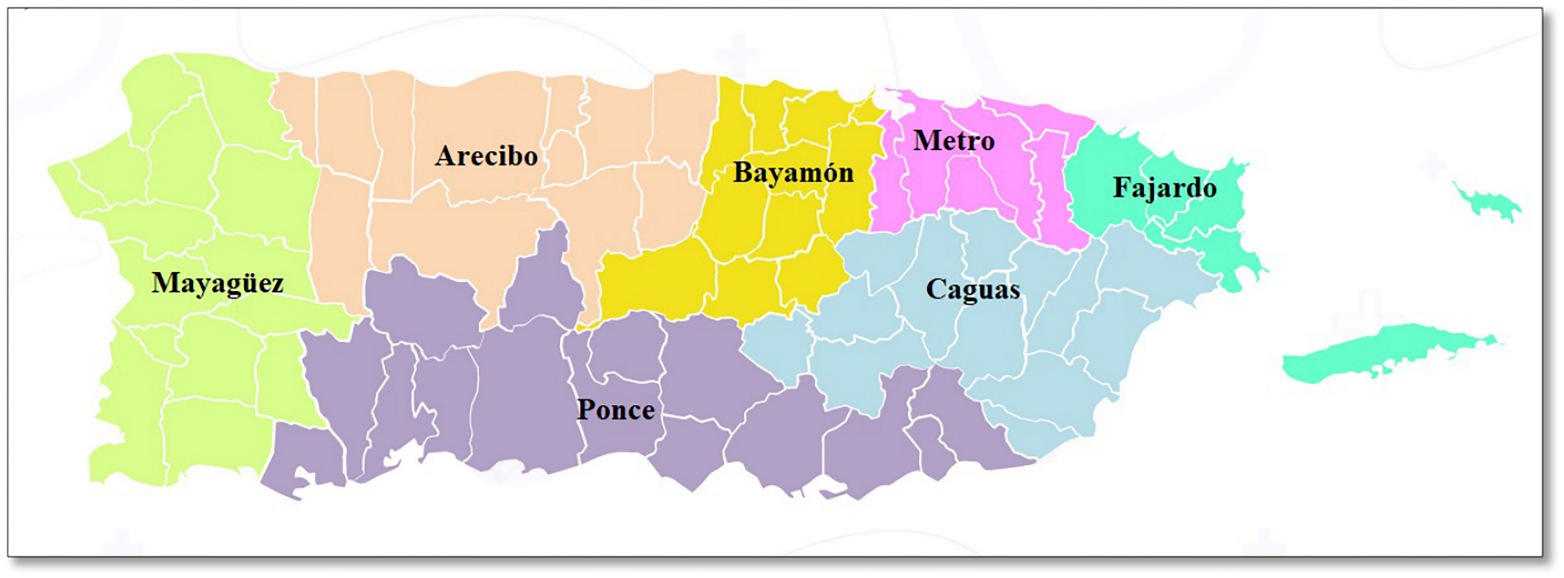
Puerto Rico geographical map by regions^1^. ^1^ Obtained and adapted from Puerto Rico Department of Health (https://www.salud.gov.pr/CMS/144).

We sent 1,427,241 messages during the study period and 26.8% were reached (Figure 1). A total of 6,975 participants completed the primary questionnaire. Table 1 shows the distribution of participants and COVID-19 cases since the lockdown in March 2020. Among the 6,975 respondents, the majority were women (68.4%) and the mean age was 51 years (SD = 14.6; range 21–93 years). Almost one-third (28.5%) of the participants were from the Metropolitan region (North), 16.5% from Caguas (Southeast), 16.2% from Bayamón (North), and 14.4% from the Ponce region (South) (Figure 2). Overall, 71.3% of the participants reported getting tested for COVID-19 since the lockdown; testing was similar for men (69.9%) and women (71.9%) (Table 1). Approximately 2.2% of participants were confirmed cases diagnosed by PCR tests, and 6.5% of the participants were confirmed/probable cases. Of the total cases, 26.2% were confirmed, 48.0% were probable based on other tests, and 25.8% were identified from the model (data not shown in tables). Women and people under 40 years of age had higher confirmed and total cases (Table 1). Arecibo and Bayamón had the highest total cases compared to other regions.

**Table 1:** Distribution of participants and COVID-19 cases since the lockdown by age, sex, and regions.

	**Total participants**	**Participants tested for COVID-19**	**Confirmed cases (PCR)**	**Confirmed and probable cases^b^**
	***N*** **(% of total)**	***N*** **(% of total for each subgroup)**		
Number^a^	6,975	4,971 (71.3)	155 (2.2)	452 (6.5)
**Age (years)**				
21–25	347 (5.2)	245 (70.6)	11 (3.2)	29 (8.4)
26–30	331 (4.9)	261 (78.9)	10 (3.0)	30 (9.1)
31–35	436 (6.5)	321 (73.6)	14 (3.2)	29 (6.7)
36–40	554 (8.2)	415 (74.9)	20 (3.6)	45 (8.1)
41–45	631 (9.4)	464 (73.5)	16 (2.5)	44 (7.0)
46–50	748 (11.1)	554 (74.1)	26 (3.5)	54 (7.2)
51–55	883 (13.1)	660 (74.8)	20 (2.3)	70 (7.9)
56–60	887 (13.2)	658 (74.2)	13 (1.5)	41 (4.6)
61–65	722 (10.7)	489 (67.8)	9 (1.3)	40 (5.5)
≥66	1,201 (17.8)	747 (62.2)	13 (1.1)	59 (4.9)
**Sex**				
Male	2,087 (29.9)	1,459 (69.9)	35 (1.7)	122 (5.9)
Female	4,769 (68.4)	3,427 (71.9)	117 (2.5)	320 (6.7)
Missing/Other	119 (1.7)	85 (71.4)	3 (2.5)	10 (8.4)
**Regions**				
Arecibo	661 (9.9)	466 (70.5)	17 (2.6)	50 (7.6)
Bayamón	1,085 (16.2)	797 (73.5)	27 (2.5)	82 (7.6)
Caguas	1,103 (16.5)	801 (72.6)	25 (2.3)	73 (6.6)
Fajardo	179 (2.7)	127 (71.0)	1 (0.6)	8 (4.5)
Mayagüez	798 (11.9)	539 (67.6)	21 (2.6)	55 (6.9)
Metro	1,905 (28.5)	1,387 (72.8)	46 (2.4)	126 (6.6)
Ponce	964 (14.4)	672 (69.7)	14 (1.5)	44 (4.6)

a The numbers may not always add to 6,975 due to missing data for some variables.

b Confirmed and probable cases (including other tests and/or models based on key symptoms) (13).

**Figure 3:**
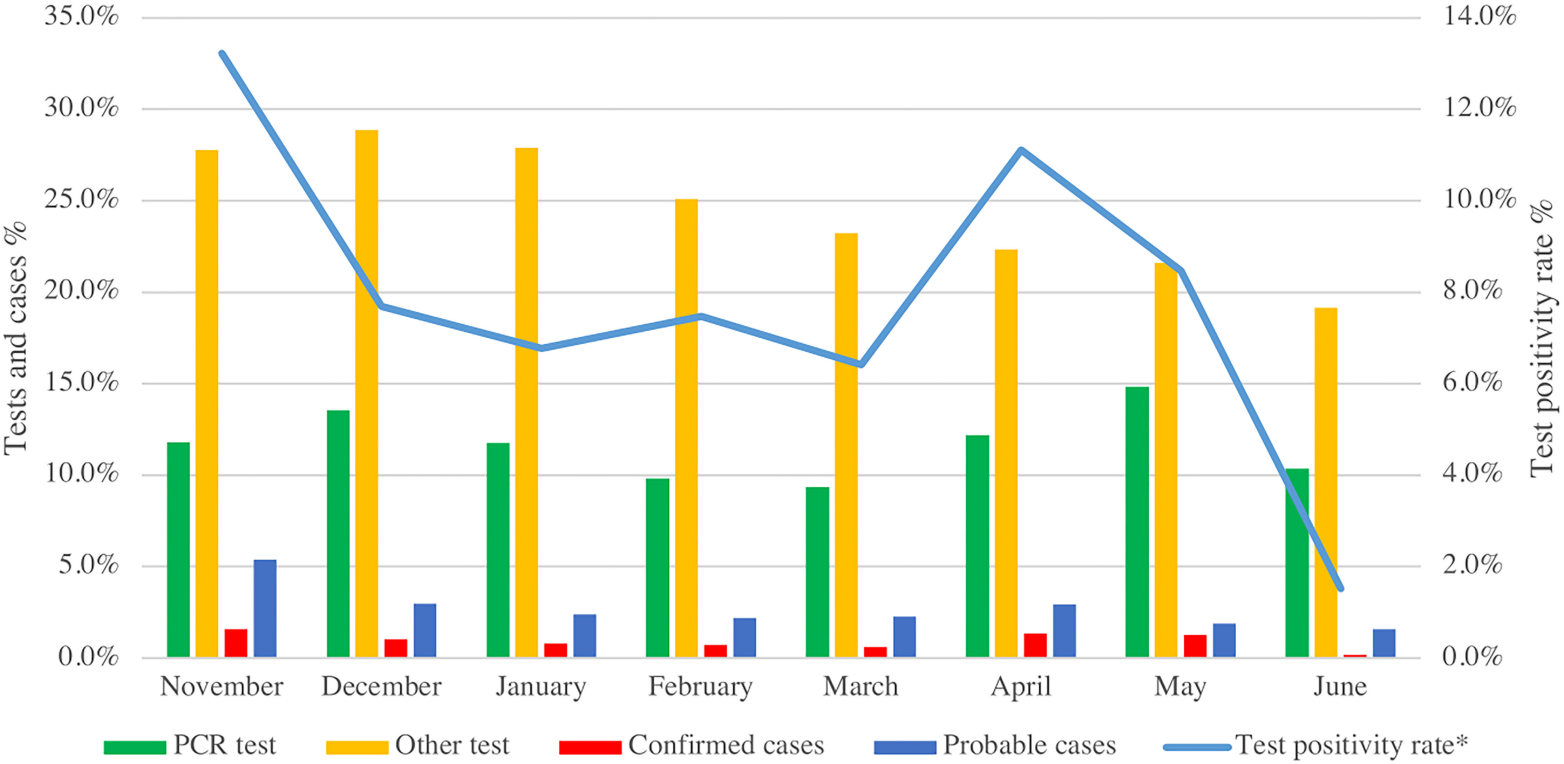
PREPCOVI testing and COVID-19 cases (confirmed and probable) distribution from November 2020 to June 2021. *Positivity rate is based on confirmed cases detected by PCR tests for SARS-CoV-2.

PREPCOVI monthly testing rates (all tests combined) ranged from 42.4% in December to 29.5% in June. The positivity rate was higher in November (13.2%) and April (11.1%) (Figure 3). From 18 October 2020 to June 2021, the test positivity rate (PCR only) was 7.9%. From November to March, the test positivity rate decreased by 6.8%. From March to April, the positivity rate increased by 4.7%, followed by a large decrease in June (positivity rate of only 1.5%). From November 2020 to June 2021, 3.6% of PREPCOVI participants were classified as cases (0.9% confirmed). Although the number of tests was higher in November, December, and January, November had the highest confirmed (1.6%) and probable (5.4%) cases. December and April had similar probable cases (3%), and June had the lowest confirmed (0.2%) and probable cases (1.6%). Women had a higher test positivity rate (9.2%) compared to men (7.7%). Positivity rates varied by age, with people aged 41–45 years having the highest positivity rate (16.4%), and people aged 61–65 years having the lowest positivity rate (3.0%) (data not shown in tables).

**Figure 4:**
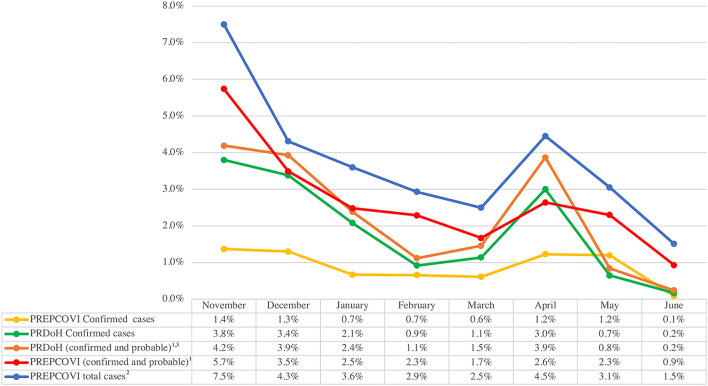
Comparison of PREPCOVI and PRDOH COVID-19 from November 2020 to June 2021 (both cases were standardized to the age and sex distribution of the PR census). ^1^ Confirmed cases are based on the PCR test, and probable cases are based on other tests. ^2^ PREPCOVI total cases include confirmed, probable, and cases based on the model using key symptoms. ^3^ PRDOH months are consistent with the test dates, whereas PREPCOVI months include the last 30 days of the survey for each participant (i.e., 1 month period for each participant, but the exact dates vary across participants and between PRDOH and PREPCOVI).

From 18 October 2020 to June 2021, PRDOH reported 71,225 confirmed cases and 83,730 total cases (confirmed and probable) among people aged 21 years or older. Since the lockdown (16 March 2020), we estimated that 6.5% of the participants had COVID-19 (2.2% confirmed and 4.3% probable, including model-based), while the PRDOH estimate was 5.3% (4.7% confirmed and 0.6% probable) for adults aged 21 years or older (data not shown in tables). The PREPCOVI total number of cases (confirmed and probable including model-based) were higher than the total number of cases reported by the PRDOH every month (Figure 4). When excluding model-based cases, PREPCOVI and PRDOH total cases were similar (higher for PREPCOVI in some months and higher for PRDOH in other months). PREPCOVI confirmed cases were lower than those reported by the PRDOH in most of the months.

**Figure 5:**
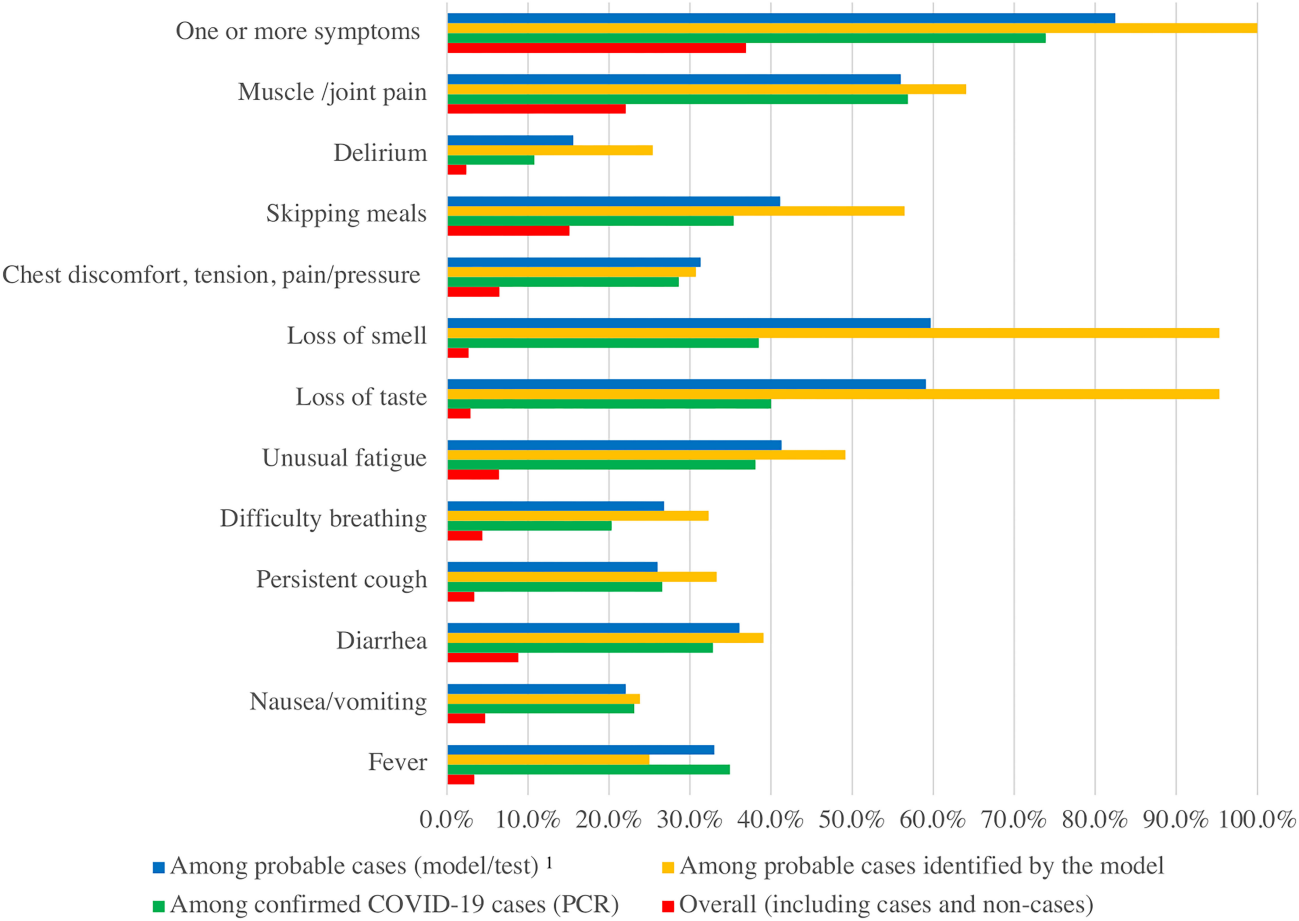
Comparison of reported symptoms by types of cases. ^1^ Probable cases (including other tests and model based on key symptoms). Only those who reported symptoms are included in the figure, hence, the numbers may not add up. People report as many symptoms as they have, hence could exceed 100%.

All participants (regardless of their COVID-19 status) were asked to report symptoms in the last 30 days (Figure 5). Among all participants (including cases and non-cases), 36.9% had one or more of the listed symptoms; muscle and/or joint pain (22.1%) was the most frequent symptom, followed by skipping meals (15.1%). Among confirmed cases, the most frequent symptoms were muscle and/or joint pain (56.9%), loss of taste (40.0%), loss of smell (38.5%), unusual fatigue (38.1%), skipping meals (35.4%), fever (34.9%), and diarrhea (32.8%). Within probable cases, loss of taste (59.1%), loss of smell (59.7%), muscle and/or joint pain (56.0%), unusual fatigue (41.3%), and skipping meals (41.1%) were the most common symptoms. Among probable cases (classified by symptoms from the published model, the majority reported the loss of taste and smell (95.3%), muscle and/or joint pain (64.1%), and skipping meals (56.5%). For all symptoms other than fever, the highest frequency was among the probable cases identified by the model.

## Discussion

More than 2 years after the first COVID-19 case was identified in China, we are still struggling with the COVID-19 pandemic. As the virus keeps mutating, new variants develop and cases plummet and skyrocket periodically despite the introduction of several vaccines. Among PREPCOVI participants, 6.5% of COVID-19 cases were identified (2.2% confirmed), compared to the PRDOH estimate of 5.3% of total cases (4.7% confirmed) in a similar period. Testing and positivity had similar time trends; in periods where positivity was higher, participants got tested more. Testing was similar across sex and regions but varied with age from 62% (≥66 years) to 79% (26–30 years). Women and younger people had the highest test positivity rate. As our data show, overall testing rates did not vary much across the study period. In April 2021, the positivity rate was very similar to November 2020, despite the availability of vaccines; this pattern was consistent in PRDOH data.

The literature shows discrepancies regarding the gender susceptibility to COVID-19. In Italy, men had higher mortality from COVID-19 compared to women, whereas women had a higher infection rate than men, which supports our findings (14). In data from PRDOH, COVID-19 total cases (confirmed and probable) were similar among men and women, while PREPCOVI data showed more cases in women. The higher mortality among men was seen consistently across China, Europe, and the United States (15). However, a large cohort from the United States showed higher infection rates in men compared to women (16).

Our data and PRDOH data show that younger participants are more likely to get COVID-19 (total cases) compared to older participants. Possible reasons could be that younger adults are more prone to high-risk situations (i.e., work settings), have a higher likelihood of attending large gatherings, and have lower vaccination compliance. Data from PRDOH show that among unvaccinated adults, a higher percentage is present among the 20–29 age group (13.9% men and 7.6% women) (17), therefore increasing the chances of COVID-19 transmission. However, older people have a higher risk of death from COVID-19 (15). PREPCOVI COVID-19 cases (confirmed and probable) were similar across regions, except in the Fajardo and Ponce regions, where we saw a lower number of COVID-19 cases, despite testing rates being similar among regions.

A total of 64 cases reported in the last 30 days (26% of total cases reported in the last 30 days) were identified from symptoms in our study that would otherwise have been missed. The published model used to classify probable cases included skipping meals, loss of taste and smell, unusual fatigue, and persistent cough (13). Confirmed cases mostly reported muscle and/or joint pain (57%), loss of taste (40%), loss of smell (39%), unusual fatigue (38%), and skipping meals (35%). Almost all the symptoms reported frequently by the confirmed cases were consistent with the model, except for muscle and/or joint pain, supporting the credibility of the model-based detection of cases. PREPCOVI data showed higher cases than the official PRDOH data, suggesting that symptom-based surveillance could be an important tool to consider adding when testing is seldom available.

This research project had several limitations. Data collection started 9 months after the first COVID-19 case was identified in Puerto Rico. This may have led to the low response rate observed since people may have felt overwhelmed and disinterested given the excessive COVID-19 information and multiple surveys. The low response rate raises some concerns about representativeness. However, participants represent 97.4% of the municipalities in PR, and our rates and trends were reasonably similar to PRDOH. Hence, we believe we have a reasonably representative sample across PR. Since participants had the option to skip questions or to refuse to answer any questions, the number of responses varied by each question, and we had varying amounts of missing data across questions. The amount of missingness is small, hence it is unlikely to bias the estimates. Also, it is important to recognize that there is a high incidence of telephone scams/voice phishing in Puerto Rico. Telecommunication companies had propaganda against this with the message: “*Don't pick-up calls of numbers you don't recognize”*, which could have generated mistrust in the population when receiving random text messages from unknown numbers despite promoting the study through different media outlets (e.g., press, social media, and radio) (18). In addition, amid a public health emergency, government agencies needed to attend to ongoing crises. Due to this and the fact that there was a change in government since we planned our study, it was difficult to engage the government in playing an active role in this project; the collaborations anticipated earlier would likely have reduced concerns among the public and increased participation rates substantially.

## Practical implications

The symptom-based model seems to be an accurate tool to classify probable cases when there is limited testing. In addition, such surveillance systems could help identify new symptoms associated with different types of variants by comparing symptoms across confirmed and probable COVID-19 cases, influenza cases, and non-cases. The PRDOH (as most entities) did not use symptom-based screening for probable cases, resulting in lower total cases than our combined approach. At the beginning of the pandemic, people were classified as suspected cases by PRDOH if they had a positive antibody test without a confirmatory PCR or antigen test. This method confused cases that could cause contagion. In addition, diagnosis based on antibody testing was even less valid after vaccination, which resulted in discontinuing the classification of suspected cases in the official reports of the PRDOH.

The world has learned the importance of testing to help prevent the spread of the virus. Despite effective vaccines, testing is still very important to address this public health crisis. In a pandemic, public health surveillance cannot stop until we are certain that the pandemic is under control. Even though symptom-based surveillance may be an alternative to identify symptomatic cases, it is challenging to identify asymptomatic cases. In addition, some researchers state that symptom-based surveillance may not be effective since the symptoms that the person presents at a given time may not provide sufficient information to correctly classify if the person has COVID-19 or another viral respiratory infection (19). Testing to confirm the infection is and will continue to be an important tool to monitor a pandemic and control spread, but testing has its limitations and challenges. We should have surveillance systems in place that can identify tested and untested, symptomatic and asymptomatic cases, and adapt to the rapidly changing nature of pandemics (i.e., changes in most common symptoms, changes due to vaccination, or availability of effective treatments).

Our data include only cases until 24 June 2021, which may not capture the subsequent waves of COVID-19 cases of the Delta and Omicron variants. Despite the early challenges and concerns, PR managed to maintain a low number of cases for several months and attained the highest vaccination rate for COVID-19 among all US states and territories (20). However, even after vaccines, more infectious variants emerged from different countries, with Omicron spreading more rapidly and with an increasing number of breakthrough infections compared to the original variant. In PR, the first case of the Delta variant was identified in June 2021 (21) while the first case of Delta plus was identified in September 2021 (22). In 2021, we saw three big waves of cases in April, August, and December (23). One of the highest daily case numbers (5,211 confirmed cases) was reported on December 23, and the highest number of reported total daily cases was 13,631 on December 27 (24).

## Data availability statement

The raw data supporting the conclusions of this article will be made available by the authors, without undue reservation.

## Ethics statement

The studies involving human participants were reviewed and approved by University of Puerto Rico, Medical Sciences Campus IRB protocol #A4840120. Written informed consent for participation was not required for this study in accordance with the national legislation and the institutional requirements.

## Author contributions

KJ is the guarantor of this study and, as such, had full access to all the data in the study, takes responsibility for the integrity of the data and the accuracy of the data analysis, conceived and designed the study, obtained funding and contributed to the analysis plan, interpretation, and writing MM-L contributed to the study design, data collection, and data analysis plan, conducted the data analyses, and wrote the initial draft of the manuscript. CV, RG, and KM contributed to the study design, data collection, and interpretation of results. WA contributed to the data collection and provided input on the interpretation. All authors read, edited, and approved the final manuscript.

## Funding

This study was supported by the University of Puerto Rico, the Puerto Rico government, and the National Institute on Minority Health and Health Disparities (U54MD007600 and S21MD001830). Funds were distributed from the General Fund of the Treasury of the Government of Puerto Rico to finance a portion of the first phase of the Strategic Plan to Reactivate Our Economy, Support Our Businesses and Protect Our Workers in response to the emergency caused by the COVID-19 pandemic. An amount of these funds was allocated to the University of Puerto Rico (UPR) Central Administration to finance research and development related to COVID-19 and related topics at the institution; part of which was awarded for this project. The Office of the UPR President did not have any role in the study design, data collection, data management, data analysis, interpretation of data, writing of the report, and the decision to submit the report for publication. The only exception was that the UPR President and the Chancellor of the Medical Sciences Campus participated in media interviews and promotions related to our study.

## Conflict of interest

The authors declare that the research was conducted in the absence of any commercial or financial relationships that could be construed as a potential conflict of interest.

## Publisher's note

All claims expressed in this article are solely those of the authors and do not necessarily represent those of their affiliated organizations, or those of the publisher, the editors and the reviewers. Any product that may be evaluated in this article, or claim that may be made by its manufacturer, is not guaranteed or endorsed by the publisher.
