# Supplementary material for: Estimating COVID-19 cases in Puerto Rico using an automated surveillance system
Source: Front Public Health. 2022 Aug 4;10:947224. doi: 10.3389/fpubh.2022.947224 (PMC9388143; doi:10.3389/fpubh.2022.947224)
Supplement: Supplementary file 1 [file Data_Sheet_1.DOCX]

**SUPPLEMENTAL MATERIAL: PREPCOVI questions pertinent to this manuscript**

1) Are you 21 years of age or older?

0. No [End questionnaire: Thank you for your interest in our study. However, we regret to inform you that we are not recruiting persons under 21 years of age.]

1. Yes [SKIP to 2]

1.A) How old are you? [“Using your number pad, please input your age”]: _ _ _

2) Are you a resident of Puerto Rico?

0. No

1. Yes

3) What is your biological sex?

1. Male

2. Female

3. Other

4) Specify your current residential 5-digit zip code [“Using your number pad, please input your current residential 5-digit zip code”]: _ _ _ _ _ _

5) In the last 30 days, how many times have you been tested for COVID-19?

0. 0 [SKIP to 5c]

1. 1

2. 2

3. 3

4. 4

5a. In the last 30 days, have you tested positive for COVID-19?

0. No [SKIP to 5c]

1. Yes

5b. In the last 30 days, what type of sample did you provide?

1. Nasal swab/Nasopharyngeal

7.b.1.a. What type of test was performed?

1. Antigen test

2. Molecular/RT-PCR

99. I do not know.

2. Blood

3. Saliva

4. Both samples (nasal swab and blood)

5. Other

5c. Since the lockdown (March 16th, 2020) how many times have you been tested for COVID-19? [SHOW IF 5=0]

0. 0 [SKIP to 6]

1. 1

2. 2

3. 3

4. 4 or more

5c1. Since the lockdown (March 16th, 2020), have you tested positive for COVID-19?

0. No [SKIP to 5d1]

1. Yes

5c2. When was the last time you tested positive for COVID-19? [SHOW IF: 5c1=1]

1. March 2020

2. April 2020

3. May 2020

4. Junio 2020

5. Julio 2020

6. Agosto 2020

7. September 2020

8. October 2020

9. November 2020

10. December 2020

11. January 2021

12. February 2021

13. March 2021

14. April 2021

15. May 2021

16. June 2021

5c3. What type of sample did you provide the last time you tested positive for COVID-19? [SHOW IF: 5c3=1]

1. Nasal swab

7c31a. What type of test was performed?

1. Antigen test

2. Molecular/RT-PCR

99. I do not know.

2. Blood

3. Saliva

4. Both samples (swab and blood)

5. Other

5d1. When was the first COVID-19 test you had performed? [SHOW IF: 5c1=0]

1. March 2020

2. April 2020

3. May 2020

4. June 2020

5. July 2020

6. August 2020

7. September 2020

8. October 2020

9. November 2020

10. December 2020

11. January 2021

12. February 2021

13. March 2021

14. April 2021

15. May 2021

16. June 2021

5e1. When was the last COVID-19 test you had performed? [SHOW IF: 5c1>1 AND 5c=0]

1. March 2020

2. April 2020

3. May 2020

4. June 2020

5. July 2020

6. August2020

7. September 2020

8. October 2020

9. November 2020

10. December 2020

11. January 2021

12. February 2021

13. March 2021

14. April 2021

15. May 2021

16. June 2021

6. Do you think you ever had COVID-19 infection? [SHOW IF: 5a=1 OR 5c=1]

0. No

1. Yes

7. In the last 30 days, have you experienced any of the following symptoms?

7a. Red and/or itchy eyes (e.g. conjunctivitis)

1. No

2. Yes

7b. Nausea and/or vomit

1. No

2. Yes

7c. Delirium (e.g. confusion, disorientation)

1. No

2. Yes

7d. Diarrhea

1. No

2. Yes

7e. Abdominal pain

1. No

2. Yes

7f. Muscle and/or joint pain

1. No

2. Yes

7g. Persistent cough (frequent coughing during more than an hour or 3 or more coughing episodes within 24 hours)

1. No

2. Yes

7h. Dry cough

1. No

2. Yes

7i. Difficulty breathing

1. No

2. Yes

7j. Fever

1. No

2. Yes

7k. Unusual fatigue

1. No

2. Yes

7l. Loss of taste

1. No

2. Yes

7m. Loss of smell

1. No

2. Yes

7n. Chest discomfort, tension, pain, and/or pressure

1. No

2. Yes

7o. Skipping meals

1. No

2. Yes

7p. Hoarse voice

0. No

1. Yes

7q. Headache

1. No

2. Yes

7r. Throat pain

0. No

1. Yes

7s. Nasal congestion and/or drip

1. No

2. Yes

7t. Chills

1. No

2. Yes
